# German Psychiatrists' Observation and Interpretation of Religiosity/Spirituality

**DOI:** 10.1155/2013/280168

**Published:** 2013-11-14

**Authors:** Eunmi Lee, Klaus Baumann

**Affiliations:** ^1^Departement of Caritas Science and Christian Social Welfare, Faculty of Theology, University of Freiburg, Platz der Universitaet 3, 79098 Freiburg, Germany; ^2^Freiburg Institute for Advanced Studies (FRIAS), Universität Freiburg, 79098 Freiburg, Germany

## Abstract

The purpose of this study was to explore how contemporary German psychiatrists think about religiosity/spirituality (ReS) in regard to their therapies. We conducted an anonymous survey among the clinical staff of psychiatry and psychotherapy departments in German university hospitals and faith-based clinics in the same cities. Two main instruments were used, the Duke University Religion Index (DUREL) and the questionnaire from Curlin et al. “Religion and Spirituality in Medicine: Physicians' Perspectives.” A total of 123 psychiatrists participated in this survey. However, due to incomplete responses, only 99 questionnaires from psychiatrists were analyzed. Results show that German psychiatrists positively experience the influence of ReS on patients' mental health. Psychiatrists' own ReS significantly influenced their interpretation of the effect of ReS on psychiatric patients as well as their attitude toward ReS in the clinical setting. The more religious psychiatrists are, the more they tend to observe a positive influence of ReS on mental health. In light of these results, psychiatrists should be aware of their own religious/spiritual characteristics and also reconsider their assumptions about professional neutrality and value openness. Furthermore, training programs on religious/spiritual issues and effective teamwork with chaplains are recommended.

## 1. Introduction

While it is common for believers who are ill to pray for healing or strength to endure the challenges of their illness, religious practices have often been viewed skeptically by psychiatric staff. In fact, several symptoms of psychological disorders can be connected with atypical or exaggerated religious/spiritual phenomena. Nevertheless, studies have shown that religion and/or spirituality are important for psychiatric patients. For example, Cunningham et al. found that Irish people with depression or bipolar disorder associate their religious/spiritual beliefs with solace and hope; especially when patients felt that they could not control their psychiatric problems, their beliefs safeguarded them against feelings of helplessness [1]. According to the reports of psychiatric patients, religiosity/spirituality (ReS) is an important part of their lives and particularly helpful in times of sickness [1–3].

In recent decades, the number of studies on the relationship between ReS and mental health has grown steadily. Research has used different traits, for example, religious affiliation, churchgoing, or personal importance, to examine the role of ReS among different populations and has shown inconsistent results. Various studies, though, have demonstrated that ReS has a positive effect on a number of psychiatric problems, including depression [4–6], suicide [7, 8], substance abuse disorder [9, 10], anxiety disorder [11], and posttraumatic stress disorder [12, 13]. One study conducted by Kim and Seidlitz with Korean university students showed that spirituality moderated the effect of stress on negative affect, and this buffer function was stronger for students with a religious affiliation [14]. Another study by Miller et al. revealed that those who consider religion and/or spirituality an essential aspect of their lives have one-fourth the risk of having a major depression than those who do not find ReS important [4]. Furthermore, those with major depressive parents who highly rated religion and/or spirituality showed one-tenth the risk of experiencing major depression than the comparison group.

Still, there are also empirical studies that have not confirmed a positive influence of ReS on psychiatric patients [15, 16]; some have even shown negative effects of ReS [17, 18]. For instance, Büssing and Mundle surveyed German patients with depressive and/or addictive disorders. According to their results, intrinsic religiosity as measured by *Reliance on God's Help* (RGH) and depression as measured by *Beck's Depression Inventory* (BDI) were not significantly associated [15].

Along with the growing body of research, there is an increase in international interest and discussions about the integration of ReS into therapeutic settings [19–21]. In addition, psychiatric patients desire that their religious/spiritual needs can be handled by their medical staff [22, 23]. Yet, psychiatrists appear less open to religious/spiritual issues in the “standard” clinical routine. For example, British psychiatrists in a study by Durà-Vilà et al. generally had a positive attitude toward ReS in psychiatry and psychotherapy, but none of them considered it part of their routine clinical practice [24]. Hence, therapeutic processes in psychiatry and psychotherapy typically do not specifically address religious/spiritual issues in the clinical setting. When such topics are discussed, patients are generally the ones who actively bring up these subjects, not their psychiatrists or psychotherapists [2].

There are plausible reasons why psychiatrists are reluctant to deal with religious/spiritual issues and/or related activities. The scientific critique of religions as such, which was greatly influenced by Sigmund Freud, may be the most prominent reason. Freud observed the similarities between obsessive-compulsive neurosis and religious rituals and/or behavior of religious persons [25, 26]. Although few contemporary psychiatrists would follow him rigidly, Freud's theory and his influence can hardly be disregarded. Another aspect is the fact that psychiatrists usually encounter phenomena of ReS in a pathological context, such as delusions or hallucinations with religious contents. In this regard, German psychiatrist Wyss doubted whether there is any “Neurosis” or “Psychosis” without some kind of distorted religious content [27, 28]. This can be seen not only in clinical practice but also in training materials, particularly in Germany [26, 29, 30]. For instance, contemporary psychiatry and psychotherapy textbooks hardly mention religious/spiritual topics; when they are mentioned, it is only in negative contexts [26, 29, 30].

In our pilot study, psychiatrists pragmatically mentioned lack of time as one of the most frequent barriers to addressing religious/spiritual issues in therapeutic processes [31]. In addition, psychiatric staff also cited their obligation to maintain professional neutrality, in the sense that patients must not be influenced by psychiatrists' own ideologies, mental attitudes, or other positions [31].

International interest and discussions about an adequate integration of ReS in therapeutic settings are growing, though not as strongly in German-speaking areas as in other countries, such as USA. Following the preliminary results of our pilot study, this survey aimed to answer the following questions: how do German psychiatrists and medical psychotherapists perceive and interpret the effect of ReS on their patients in hospital settings? What makes them reluctant to commonly integrate ReS into their therapies?

## 2. Materials and Method

### 2.1. Respondents

An anonymous survey was conducted from October 2010 to February 2011 to explore the viewpoints of psychiatric staff in regard to ReS. Psychiatric staff in this study was medical, (psycho-) therapeutic, and nursing staff working directly with patients. The survey involved clinical staff from psychiatry and psychotherapy departments in German university hospitals and faith-based clinics in the same cities. Overall, 12 of 32 university hospitals and 9 of 21 faith-based clinics participated in this survey.

The medical director of each psychiatry/psychotherapy department distributed a paper-based questionnaire to psychiatric staff. Of 1,654 distributed questionnaires, 404 were returned (response rate = 24.43%). A total of 123 questionnaires (32%) had been filled out by psychiatrists. For the purpose of our analysis, we focused only on the psychiatrists. An isolated response rate among the psychiatrists could not be calculated, as only the total number of psychiatric staff in each hospital could be obtained at the beginning of the survey. Due to incomplete responses, only 99 questionnaires from psychiatrists were analyzed.

### 2.2. Measures

We operationalized ReS by implementing two measures, the Duke University Religion Index (DUREL) and a questionnaire on “Religion and Spirituality in Medicine: Physicians' Perspectives” developed by Curlin et al. [32]. Each instrument was used to measure psychiatrists' religious/spiritual characteristics, their observation/interpretation of the influence of ReS on patients' mental health, and also their attitudes/self-reported behavior toward ReS in therapeutic settings.

Using these two instruments, a pilot study was conducted in the department of psychiatry and psychotherapy of the Freiburg University Hospital in Germany from December 2008 to January 2009 [31]. Prior to the pilot study, these instruments were translated into German (for the first time) and revised by a team of professionals.

#### 2.2.1. DUREL

DUREL, developed by Koenig et al., is a widely accepted and well-known instrument for measuring basic religious/spiritual traits. Using DUREL, we measured organizational religiosity by asking the question “How often do you attend church or religious meetings?”, measured according to a 6-point scale with response options ranging from *more than once a week* to *never. *Nonorganizational religiosity was measured by asking the question “How often do you spend time in private religious activities, such as prayer, meditation, or Bible study?”, using a 6-point scale with response options ranging from *more than once a day* to *rarely or never*. DUREL also incorporated three questions to measure intrinsic religiosity, which we combined into one item for our analyses: “My religious beliefs are what really lie behind my whole approach to life,” “I try hard to carry my religion over into all other dealings in life,” and “In my life, I experience the presence of the Divine (i.e., God).” These three questions were measured according to a 5-point scale, with response options ranging from *definitely not true* to *definitely true of me*. The three items related to intrinsic religiosity were originally obtained from Hoge's 10-item Intrinsic Religiosity Scale and strongly correlated with Hoge's original items (*r* = .85); reliability (*α* = .75) was demonstrated for these three items as well [33, 34]. In our study, the three items of intrinsic religiosity also showed a strong reliability (internal consistency) of *α* = .911.

#### 2.2.2. Curlin et al. Questionnaire

Curlin and colleagues developed a questionnaire to measure physicians' observations and interpretations of the influence of ReS on patients' health as well as their attitudes and self-reported behaviors regarding religious/spiritual issues in clinical settings. This questionnaire was developed by several qualitative pilot interviews and was tested via multiple iterations of expert panel review [32].

Based on comments from respondents in the pilot study, response options of each category were modified to a 5-point scale. The category regarding physicians' observations/interpretations was transformed into a 5-point scale (1: never, 5: always); likewise, the category regarding physicians' attitudes/self-reported behaviors was transformed into a 5-point scale (1: definitely not true, 5: definitely true of me). All items were redesigned into statements rather than questions. In addition, we decided to use the expression “religiosity/spirituality” rather than the original terminology “religion/spirituality.” This was intended to encompass all related religious/spiritual issues; in German, the term “religion” can be limited to a religious affiliation. Fully described items are listed in Tables 3 and 4.

### 2.3. Statistical Analysis

Data was evaluated with SPSS 20.0 for Windows. To exam the difference between groups and variables, cross-tabulation as well as Pearson-square-test, univariate analyses of variance (UNIANOVA), and Spearman's rank correlation were used. Significance level was set at *P* < .05.

## 3. Results

### 3.1. Characteristics of Survey Respondents

Roughly two-thirds of responding psychiatrists worked in university hospitals. Among the respondents, 54.5% were men and 45.5% women. On average, participants were 39.03 (SD = 8.34 (all numeric results were rounded off to the nearest hundredth.)) years old (Table 1).

While about 57% considered themselves a believing person, 70.7% reported a religious affiliation. Nearly 41% of the responding psychiatrists indicated attending church or religious meetings once a year or less, and only 10.1% reported going to a religious service at least once a week. In regard to private religious activities like prayer, meditation, or scripture reading, more than half of the participants responded that they rarely or never spent their time on such activities; nevertheless, close to 30% reported doing so several times per week.

Slightly over half of the respondents agreed with the statement that their religious beliefs are central for their whole approach to life, and about 37% agreed that they try to carry their religion over into all other parts of their life. About 36% of the participants responded that they had experienced God or a higher being. The detailed results are described in Table 2.

Moreover, the score of intrinsic religiosity was calculated as the sum of the three items, whereupon *m* = 6.71 (SD = 3.07, *N* = 79) on a scale of 12.0 (To ensure that the nature of the ordinal scale was not affected in the German version, the translated answer “unsure” was removed in the analysis of the sum of intrinsic religiosity, as the German word can mean either “I am not sure” or “I have no idea.” The highest possible score was therefore 12.0 rather than 15.0, and an intrinsic religiosity score could be calculated for 79 cases. Furthermore, we tested to see if there were any significant differences related to the response option “unsure” according to demographic characteristics (clinic, sex, age and religious affiliation). However, no significant differences were found.) (3.0 lowest value to 12.0 highest value). This was marginally lower than the median. Concerning subgroups (clinic, sex, and age), the differences in intrinsic religiosity scores were compared using UNIANOVA. It is noteworthy that no significant differences were found for any of the three of these supposedly distinctive variables.

### 3.2. Psychiatrists' Observations and Interpretations of the Influence of ReS on Patients' Mental Health

In the clinical setting, psychiatrists seem to be fairly frequently confronted with ReS and often even quite positively (data not shown). Approximately 57% of psychiatrists reported that their patients sometimes mentioned religious/spiritual issues, and 20% encountered such topics often. About 54% of the participants often observed that ReS helps patients to cope with their illness, and 42% observed this sometimes. Most of the respondents experienced that ReS supports a positive, hopeful state of mind in their patients (52.5% sometimes and 41.4% often). More than 70% did not observe that their patients refuse medically indicated therapy or avoid taking responsibility for their health status because of their religious/spiritual attitudes.

Is psychiatrists' own ReS associated with the way they observe and/or interpret the influence of ReS on patients? Significant correlations were found (Table 3); as the intrinsic religiosity scores of the psychiatrists increased, so did their perception of the positive effects of ReS. For example, the more religious psychiatrists are, the more they tend to observe a generally positive influence of ReS on mental health (*r* = .418, *P* < .0001) and the more they believe that ReS helps psychiatric patients to endure their illness (*r* = .388,   *P* < .0001). The only item that did not significantly correlate with psychiatrists' intrinsic religiosity was to what degree they think that suffering from an illness often leads patients to ReS (*r* = .073,   *P* = .262).

Again, in contrast to common sense expectations, there was no difference between faith-based clinics and university hospitals as to the physicians' observations and interpretations of the influence of ReS on patients' mental health.

### 3.3. Psychiatrists' Attitudes and Self-Reported Behavior regarding ReS in Clinical Settings

More than 75% of psychiatrists in our sample found it appropriate to ask about religion and/or spirituality, and more than 90% found the discussion of religious/spiritual issues appropriate when patients bring them up (data not shown). Listening carefully and empathetically to religious/spiritual topics was affirmed by 98% of the respondents; about 82% reported not turning away from such topics, and nearly 80% indicated that they encourage their patients to practice their religious/spiritual activities. When religious/spiritual topics enter the dialogue, 59.6% of psychiatrists responded that they prefer to refer their patients to chaplains, and 16.2% answered that they would definitely do so.

Generally speaking, psychiatrists seem reluctant to take part in religious/spiritual activities within their professional or clinical contexts. About 80% indicated that it is not appropriate to share their own ReS with patients, and only 12.1% of them reported actually doing so (11 of 99 shared their beliefs to some extent, and one respondent invariably did so). Praying with patients was perceived particularly critically. More than 90% regarded it as an improper act, and again only one respondent reported actually praying with patients.

Again, significant but weak correlations were shown between the psychiatrists' own ReS and their attitudes as well as self-reported behaviors toward ReS in therapeutic settings (Table 4) (To ensure that the nature of the ordinal scale was not affected in the German version, the translated answer “unsure” was removed in the analysis of the sum of intrinsic religiosity, as the German word can mean either “I am not sure” or “I have no idea”. Furthermore, we tested to see if there were any significant differences related to the response option “unsure” according to demographic characteristics (clinic, sex, age and religious affiliation). However, no significant differences were found.). Psychiatrists with higher intrinsic religiosity scores found the integration of ReS more appropriate and also reported more positive behaviors toward ReS. For instance, the more religious psychiatrists are, the more they tend to consider prayer as a potentially appropriate intervention (*r* = .382, *P* < .0001) or actually pray together with patients (*r* = .281, *P* = .006). Yet, there was no correlation between psychiatrists' own ReS and their readiness to discuss religious/spiritual issues with patients (*r* = .135, *P* = .123) or to refer patients to chaplains (*r* = .063, *P* = .301). Psychiatrists in faith-based clinics reported more often than those in university hospitals that they refer their patients to chaplains, when ReS issues come up in discussion with patients (*m* = 3.19 versus *m* = 2.83, *P* = .022). This is the only significant difference between the tested variables of psychiatrists of the two types of clinics.

**Figure 1:**
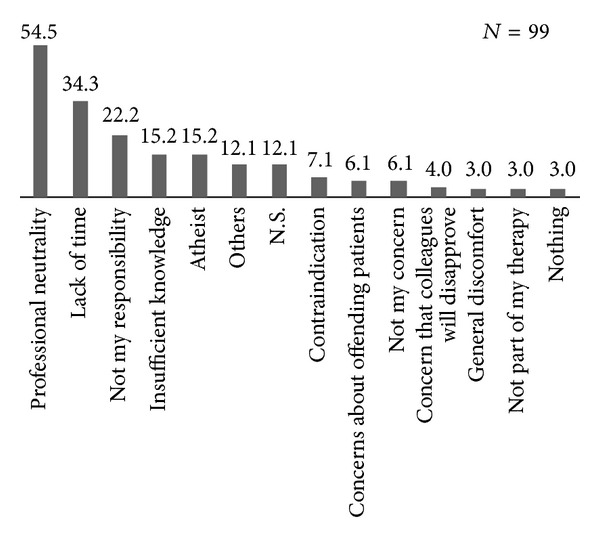
Barriers to the integration of ReS.

We also asked psychiatrists what kind of obstacles might make it difficult to integrate ReS into their therapeutic work (Figure 1). It was possible to give multiple answers. The most frequently mentioned barriers were professional neutrality (54.5%) and lack of time (34.3%), followed by the opinion that it is not psychiatrists' responsibility (22.2%).

To conclude, psychiatrists were asked whether they regard ReS as a coping strategy and whether ReS could aggravate or even cause psychiatric disorders. Most of the respondents considered ReS a coping strategy (54.5% sometimes and 34.3% often, data not shown). About 70% answered that ReS can aggravate mental health problems (62.6% sometimes and 9.1% often) but do not usually think that it causes psychiatric disorders (23.2% never and 48.5% rarely). Again, the psychiatrists' intrinsic religiosity scores were significantly but not strongly correlated with considering ReS as a coping strategy (*r* = .386, *P* < .0001).

## 4. Discussion

The present study examined how contemporary German psychiatrists in psychiatry and psychotherapy departments in university hospitals and some faith-based clinics observe and interpret ReS in their clinical practice. According to the results, psychiatrists in hospitals generally observe that ReS has a positive influence on the health of their patients. ReS can be used as a coping strategy or also provide support, especially from religious/spiritual communities. The psychiatrists in our sample claim that they are quite open to ReS issues. When patients bring up religious/spiritual concerns, psychiatrists are ready to listen and discuss these issues with them and also cooperate with chaplains. Only one respondent said he felt uncomfortable to deal with ReS matters and therefore also difficult to integrate such topics into his therapies.

Nevertheless, it is too early to say that psychiatrists in Germany are ready to actively and consistently integrate ReS into their clinical practice. They prefer to let patients address ReS issues and are open to listening in such cases; they support patients carrying out religious/spiritual practices on their own but are hesitant to engage actively in religious/spiritual matters with their patients. This is quite similar to what research shows among American psychiatrists [32]. However, differences exist with regard to “active” behaviors like asking about religious/spiritual or other personal beliefs in the anamnesis and most evidently regarding prayer. About 70% of American psychiatrists find it appropriate to pray with patients when their patients are willing and/or they find it necessary [32]. Yet, 90% of German psychiatrists considered it inappropriate to pray with patients or share their own religious/spiritual backgrounds or beliefs.

Particularly interesting is the fact that there was a significant association between psychiatrists' own ReS and their attitudes and behaviors toward their patients' ReS. Significant but weak correlations (around *r* = .3) showed that the responding psychiatrists recognize and encourage positive sides of their patients' ReS when ReS is also important in their own lives. This tendency was also found with regard to their attitudes and self-reported behaviors. Psychiatrists are more eager to integrate religious/spiritual topics and/or activities into their clinical practice when ReS also plays a role in the lives of the psychiatrists themselves. This result has also been confirmed in other recent empirical research publications [31, 32, 35]. Noteworthily, there was no significant difference except referral to chaplains between psychiatrists working in university or faith-based hospitals.

The reported results provide empirical evidence that psychiatrists' manner of dealing with patients is not unaffected by their own ideologies, worldviews, or philosophies of life. In practice, psychiatrists aim to work with a professionally neutral attitude without following unconscious tendencies (especially biases), due to transference and countertransference dynamics. The attempt to maintain neutrality is mirrored in the results of our survey, in which 54.5% of the psychiatrists indicated that professional neutrality prevents them from addressing ReS topics. The results of our survey suggest that one's own religious/spiritual beliefs and attitudes should not be disregarded; professional “neutrality” requires psychiatrists to work through their own experience, attitudes, and values in order to consciously, reflectively integrate them into their clinical practice for the benefit of their patients. Like other personal attributes such as gender, race, or political views, religious/spiritual backgrounds do affect psychiatrists' therapeutic practices [36]. In conclusion, psychiatrists need to better understand their conscious and unconscious dynamics toward ReS and how their viewpoints influence their clinical practice. Training programs should include religious/spiritual issues in the context of psychiatry and psychotherapy, as well as increased interdisciplinary teamwork with chaplains or other psychiatrists who are familiar with religious/spiritual issues. This might enrich psychiatrists' day to day life and practice and also benefit the patients.

In spite of these meaningful results and discussions, this study has several limitations worth considering. First of all, the caution is warranted when generalizing these results, as this survey was conducted in psychiatry and psychotherapy departments of university hospitals and faith-based clinics, which may not be representative of all German psychiatrists. Besides, the sample size was rather small. Additionally, the original survey was aimed at all groups of psychiatric staff, but here only the data of psychiatrists was extracted for this focused analysis. Therefore, an exact response rate of psychiatrists alone from all the participating clinics could not be calculated. Moreover, minor content differences due to the translation of the original American version into German cannot be ignored. In this sense, different cultural and religious backgrounds between the USA and Germany have been reflected in our translated version. Lastly, we measured psychiatrists' ReS using the DUREL. ReS is a broad and somewhat vague construct. There may be important aspects of ReS that the DUREL does not capture.

Future research should aim to include psychosomatic departments, private clinics, and resident psychiatrists with a large sample size for an even more representative picture. In addition, subsequent studies are needed to explore whether and how the integration of religious/spiritual elements into therapeutic processes affects therapeutic outcomes.

## 5. Conclusions

In conclusion, this survey shows the trend that contemporary German psychiatrists positively regard and interpret the influence of patients' ReS on mental health. In addition, this study indicates that psychiatrists' own religious/spiritual characteristics can affect therapeutic processes to a significant extent. Without being aware of it, psychiatrists' own ReS and attitudes toward ReS influence the extent to which they integrate the religious/spiritual needs of their patients into their therapies. In the light of these results, it is recommended that psychiatrists be aware of their own religious/spiritual experiences and attitudes. Furthermore, training programs dealing with ReS and effective interdisciplinary work with chaplains would be helpful to handle patients' ReS more suitably.

## Figures and Tables

**Table 1:** Characteristics of survey respondents.

Variable	Values (%)
Absolute number	99
Age (years)	39.03 ± 8.34
Clinic	
University hospitals	64 (64.6)
Faith-based clinics	35 (35.4)
Sex	
Female	45 (45.5)
Male	54 (54.5)
Denomination	
Have a religious affiliation	70 (70.7)
No religious affiliation^a^	29 (29.3)
Self-expression as a…	
Believing person	56 (56.6)
Nonbelieving person	43 (43.4)
Church attendance	
More than once a week	2 (2.0)
Once a week	8 (8.1)
A few times a month	14 (14.1)
A few times a year	35 (35.4)
Once a year or less	25 (25.3)
Never	15 (15.2)
Private religious activities	
More than once a day	3 (3.0)
Daily	13 (13.1)
Two or more times per week	13 (13.1)
Once a week	6 (6.1)
A few times a month	12 (12.1)
Rarely or never	52 (52.5)

^a^Atheist, agnostic, and none.

**Table 2:** Psychiatrists' intrinsic religiosity.

	Definitely true of me	Tends to be true	Unsure	Tends not to be true	Definitely not true
Religious beliefs influence whole approach to life	19 (19.2)	34 (34.3)	3 (3.0)	18 (18.2)	25 (25.3)
Try to carry religion into other aspects of life	7 (7.1)	30 (30.3)	6 (6.1)	26 (26.3)	30 (30.3)
Experience God's presence	14 (14.1)	22 (22.2)	16 (16.2)	15 (15.2)	32 (32.3)

**Table 3:** Psychiatrists' observations and interpretations of the influence of ReS on patients' health.

Questionnaire Items^a^	Analysis	
	Mean^b^	Correlation with intrinsic religiosity^c,d^
*Mention of religiosity/spirituality *		
Patients mentioned ReS issues such as God, prayer, meditation, the Bible, and so forth.	2.96 ± 0.68	0.225*
*Positive influence of religiosity/spirituality *		
The influence of ReS on health is generally positive.	3.14 ± 0.73	0.418***
ReS helps patients to cope with and endure illness.	3.52 ± 0.61	0.388***
Patients have received emotional or practical support from their religious community.	3.20 ± 0.71	0.229*
ReS gives patients a positive, hopeful state of mind.	3.34 ± 0.63	0.374***
ReS helps patients to prevent “hard” medical outcomes like death via suicide.	3.11 ± 0.75	0.301**
Suffering from an illness often leads patients to ReS.^e^	2.84 ± 0.65	0.073
*Negative influence of religiosity/spirituality *		
ReS leads patients to refuse, delay, or stop medically indicated therapy.	2.25 ± 0.63	−0.301**
Patients used ReS as a reason to avoid taking responsibility for their own health.	2.13 ± 0.62	−0.337**

^a^Preceded by “considering your experience….”

^b^Response categories are 1 = never, 2 = rarely, 3 = sometimes, 4 = often, and 5 = always.

^c^Correlation between the sum of psychiatrists' own intrinsic religiosity scores and their response to the items.

^d^Spearman's correlation (1 tailed): ***P < 0.001, **P < 0.01, *P < 0.05.

^e^In the original questionnaire, this item asked whether “religiosity/spirituality causes guilt, anxiety, or other negative emotions that lead to increased patient suffering” and belonged to the category: negative influence. Based on comments from the respondents of the pilot study and other comments from a professional team, this question was replaced by the item “Suffering from an illness often leads patients to religiosity/spirituality.”

**Table 4:** Psychiatrists' attitudes and self-reported behaviors regarding ReS in clinical settings.

Questionnaire items	Analysis	
	Mean^a^	Correlation with intrinsic religiosity^b,c^
*Attitudes *		
In general, it is appropriate for a psychiatrist to inquire about a patient's religion and/or spirituality.	3.18 ± 0.83	0.243*
In general, it is appropriate for a psychiatrist to discuss religious/spiritual issues, when a patient brings them up.	3.47 ± 0.63	0.135
In general, it is appropriate for a psychiatrist to talk about his or her own religious beliefs or experiences with a patient.	1.73 ± 0.75	0.281**
In general, it is appropriate for a psychiatrist to pray with a patient together.	1.30 ± 0.51	0.382***
*Self-reported behaviors* ^ d^		
I listen carefully and empathetically.	3.76 ± 0.46	0.242*
I try to change the subject in a tactful way.	1.82 ± 0.79	−0.273**
I encourage patients in their own religious/spiritual beliefs and practices.	3.18 ± 0.65	0.228*
I respectfully share my own religious ideas and experiences.	1.58 ± 0.73	0.332**
I pray with the patient.	1.12 ± 0.36	0.281**
I refer patients to chaplains.	2.96 ± 0.72	0.063
It is not my responsibility.	1.76 ± 0.86	−0.326**

^a^Response categories are 1 = definitely not true, 2 = tends not to be true, 3 = tends to be true, and 4 = definitely true of me.

^b^Correlation between the sum of psychiatrists' own intrinsic religiosity scores and their response to the items.

^c^Spearman's correlation (1 tailed): ***P < 0.001, **P < 0.01, *P < 0.05.

^d^Preceded by “when religious/spiritual issues come up in discussions with patients.”
